# Liquid Crystal Elastomer-Based Magnetic Composite Films for Reconfigurable Shape-Morphing Soft Miniature Machines

**DOI:** 10.1002/adma.202006191

**Published:** 2021-01-14

**Authors:** Jiachen Zhang, Yubing Guo, Wenqi Hu, Ren Hao Soon, Zoey S. Davidson, Metin Sitti

**Affiliations:** Physical Intelligence Department, Max Planck Institute for Intelligent Systems, Stuttgart 70569, Germany; Physical Intelligence Department, Max Planck Institute for Intelligent Systems, Stuttgart 70569, Germany; Physical Intelligence Department, Max Planck Institute for Intelligent Systems, Stuttgart 70569, Germany; Physical Intelligence Department, Max Planck Institute for Intelligent Systems, Stuttgart 70569, Germany; Physical Intelligence Department, Max Planck Institute for Intelligent Systems, Stuttgart 70569, Germany; Physical Intelligence Department, Max Planck Institute for Intelligent Systems, Stuttgart 70569, Germany; Department of Information Technology and Electrical Engineering, ETH Zürich, ETH Zürich, Zürich 8092, Switzerland

**Keywords:** liquid crystal elastomers, magnetic composites, programmable shapemorphing, soft machines, stimuli-responsive materials

## Abstract

Stimuli-responsive and active materials promise radical advances for many applications. In particular, soft magnetic materials offer precise, fast, and wireless actuation together with versatile functionality, while liquid crystal elastomers (LCEs) are capable of large reversible and programmable shapemorphing with high work densities in response to various environmental stimuli, e.g., temperature, light, and chemical solutions. Integrating the orthogonal stimuli-responsiveness of these two kinds of active materials could potentially enable new functionalities and future applications. Here, magnetic microparticles (MMPs) are embedded into an LCE film to take the respective advantages of both materials without compromising their independent stimuli-responsiveness. This composite material enables reconfigurable magnetic soft miniature machines that can self-adapt to a changing environment. In particular, a miniature soft robot that can autonomously alter its locomotion mode when it moves from air to hot liquid, a vine-like filament that can sense and twine around a support, and a light-switchable magnetic spring are demonstrated. The integration of LCEs and MMPs into monolithic structures introduces a new dimension in the design of soft machines and thus greatly enhances their use in applications in complex environments, especially for miniature soft robots, which are self-adaptable to environmental changes while being remotely controllable.

Stimuli-responsive materials have been intensively investigated due to their promising applications in many fields with radically improved performance compared with conventional counterparts. Active materials that mechanically respond to external stimuli, e.g., light,^|1–4]^ heat,^[5–8]^ and magnetic field,^[9–14]^ via shape-morphing, show great potential in novel actuators. In particular, liquid crystal elastomers (LCEs) stand out from others due to the programmable, reversible, and large mechanical deformation capabilities induced by multiple external inputs, including light,^[1–4]^ heat,^[5–8]^ chemical solvent,^[15]^ and electric fields. I^[16]^ LCEs are soft crosslinked polymers composed of anisotropic molecules called mesogens with predesigned molecular orientations. In the ordered state, the director field $\vec{n}$ describes the coarse-grained alignment of the mesogens. When stimulated, the mesogens change from an ordered (nematic) state to a disordered (isotropic) state and generate bulk contracting and expanding strains parallel to and perpendicular to the director field $\vec{n}$, respectively.^[17]^ The concurrent emergence of contracting and expanding strains interplays with the bulk geometry to enable 2D and 3D shapemorphing, unlike other active materials, e.g., hydrogels^[18]^ and shape memory poly-mers,^[19–21]^ which require additional passive layers for shapemorphing. LCE is also superior to other responsive materials in terms of its optical characteristics^[22]^ and high work densities with unusual force-displacement characteristics,^[6,8,23,24]^ where 2D-to-2D,^[17,25]^ 2D-to-3D,^[26,27]^ and 3D-to-3D shape-transformations^[15,28–35]^ of LCE materials have been demonstrated. These capabilities and advantages of LCEs have been utilized to demonstrate actuators for a variety of soft-bodied robots^[1,4,7,16,31–35]^and bioinspired devices, such as an artificial iris,^[2,36]^ an artificial aquatic polyp,^[37]^ and a microwalker.^[32]^ Integrating other functional components, e.g., liquid metals (LMs),^[38,39]^ into LCEs is a promising route to create additional versatile smart materials for new applications.

Meanwhile, magnetically responsive soft composites have enabled wireless milli- and micrometer-scale soft robots with promising potential in fields like medicine, bioengineering, and desktop manufacturing.^[9,13,40]^ With miniaturized deformable bodies, magnetically actuated soft robots can access small and constrained environments that are hard-to-reach and impossible for other larger and rigid devices and are safer as medical devices with significantly reduced device-tissue interaction forces. Compared with other stimuli, magnetic fields are safe, precise, fast, and easy to generate and manipulate, and can simultaneously, instantaneously, and remotely exert forces as well as torques on the same object.^[9,10,13,14,40]^ Compared with other external inputs, such as light, the strength, direction and spatiotemporal variations of magnetic fields could be easily and accurately controlled, offering much better versatility and controllability. Most of the reported robots only demonstrate one mode of activation and cannot be reconfigured in situ or adapt to a changing environment, although efforts have been made to achieve multiple functionalities with^[41–43]^ and without^[13]^ in situ reconfiguration. Existing soft robots often rely on only one kind of intrinsically deterministic stimuli-responsive soft material. Re-magnetization has been proposed that could change the magnetization profile of a device in situ,^[44]^ but only preliminary proof-of-concept results have been shown to date on this topic. Furthermore, existing magnetic devices lack the sensitivity to environmental cues and fall short on adaptability.

Here, we demonstrate a soft monolithic composite film that utilizes LCE as its base soft matrix and integrates MMPs inside for remote magnetic controllability that enables untethered in situ reconfigurable soft miniature machines. This multi-responsive film can be controlled by magnetic fields and exhibits the functionalities and versatilities similar to previously reported magnetic materials.^[13]^ It also incorporates LCE responsiveness to environmental stimuli, e.g., temperature variation and UV light illumination, resulting in multiple degrees of freedom (DOF) control that enables multi-responsive, multifunctional, and reconfigurable soft machines. The machines made from the composite change their overall shapes in situ to adapt to changing environments and exhibit multiple functionalities. Previous studies have integrated magnetic-responsiveness into LCEs via bilayer^[45,46]^ and monolithic approaches.^[4,47–51]^ However, they neglected the most prominent advantages of employing magnetic fields as an independent actuation and control signal for shape-morphing controllability and locomotion functionalities. Here, we demonstrate a miniature soft robot made from the reported material that shows multi-modal locomotion in different working environments, i.e., air and viscous liquid, an environment-sensitive filament mimicking vine twining, and a bistable in situ reconfigurable magnetic spring.

The reported material, which is a composite film, is schematically illustrated in Figure 1a, together with an example director field $\vec{n}$ and the chemical structures of the LCE base matrix and a photoresponsive component, which gave the film a reddish tint. We prepared samples with 40 μm thickness and a circular director field $\vec{n}$ to illustrate the concept of an aligned LCE composite film. Here, the highest programmed thickness of the LCE film is limited to 100 μm due to the limited anchoring strength of the polyimide-coated and rubbed surface.^[8]^ By setting the MMP-to-LCE mass ratio to be 0:1, 1:4, 1:2, 1:1, and 2:1, we varied the MMP volume ratios as 0%, 3.8%, 73%, 14%, and 24%, respectively. We examined these composites between crossed polarizers and with optical microscopy at different temperatures, as shown in Figure 1b. The observations made between crossed polarizers clearly showed that the patterning of the base LCE matrix was maintained at least until the MMPs were dense enough to completely block the light path. We observed these samples in water baths at 25 and 70 °C. All samples underwent out-of-plane shape-morphing when exposed to the high temperature bath, even the one with the highest MMP concentration. However, the shape-morphing behavior of the films with an increased MMP concentration, i.e., the mass ratio of 1:1 and 2:1, deviated more from the anticipated pure LCE response, i.e., the out-of-plane cone shape.

Though the presence of MMPs may have disturbed the director field locally, we suspect that, up to a very high MMP concentration, the LCE actuation behavior did not degrade much because the LCE maintained the programmed director field in areas without MMPs, and the programmed surface alignment penetrated some depth into LCE in areas with MMPs. Polarized optical microscopy images of a uniformly aligned LCE film with moderate concentrations (73% v/v) of embedded MMPs (Figures S1 and S2, Supporting Information) demonstrated that the LCE director field was maintained in areas without MMPs. In areas with MMPs, the programmed surface alignment created elastic repulsion between the MMPs and the top and bottom surfaces, which concentrated the particles near the middle of the film thickness,^[52]^ and is consistent with our observations from cross-sectional scanning electron microscope (SEM) images in Figure 1b. Even at a very high MMP concentration (24% v/v), the observed shape-morphing behaviors may be attributed to the fact that the global director field $\vec{n}$, to some extent, still remained intact, leading to out-of-plane actuation triggered by high temperature. Overall, the introduction of MMPs into the LCE matrix at moderate mass ratios did not entirely destroy the programmed alignment of mesogens and the director field $\vec{n}$, and thus, the composite film exhibited stimuli-responsiveness comparable to a pure LCE. Additional characterizations of the crosslinking of the material are included in Figure S3 in the Supporting Information.

We characterized the effect of MMPs on the LCE phase behavior by measuring the heating and cooling transition temperature (*T*_NI_) between the nematic and isotropic state of the composite material at different MMP concentrations. We prepared non-polymerized samples of 40 μm thickness and a uniform director field configuration with different mass ratios (MMPs:LCE) at 0:1, 1:4, 1:2, 1:1, and 2:1. We tested the samples in both a rising temperature and a falling temperature scenarios by observing the microscopic textures of samples placed in a heating chamber assembled on an optical microscope stage. The heating and cooling rates were set as 5 °C per minute. The addition of MMPs did not have a large impact on the value of T_NI_ of the composite material (Figure 2a).

We then investigated the formation of helices^[53]^ induced by the shape-morphing of the composite films with a splay director field $\vec{n}$ in response to temperature changes (Figure 2b). We prepared samples of a 25 μm thick film at 1:2 mass ratio (MMPs:LCE) with a dimension of 1 × 9 mm^2^ via laser cutting them at a 45° angle with the director field $\vec{n}$ of the film. We submerged the samples in liquid (glycerol, ≥99.5%, Sigma-Aldrich, Merck KGaA) to ensure a homogeneous temperature around them. The samples formed helical shapes with increased temperature, as expected for this director field configuration and geometry in pure (non-composite) LCE. In the composite film above the actuation temperature, we observed (Figure 2b) a roughly linear relationship between the number of helical turns and the applied temperature. The strain characterization of uniform aligned LCE films with different concentrations of MMPs is presented in Figure S4 (Supporting Information). A further characterization of the material at temperatures beyond the designed working range (up to 300 °C) is available in Figure S5 in the Supporting Information.

In addition to the shape-morphing capabilities imparted by the LCE soft matrix, the composite film exhibited magnetic characteristics endowed by the embedded MMPs. These magnetic characteristics enabled an independent actuation modality and DOF for controlling the film shape-morphing besides the thermal and light stimuli-responsiveness of the LCE base matrix. We characterized the magnetic properties of the composite using a vibrating sample magnetometer (VSM, EZ7, Microsense). The four-quadrant and initial hysteresis measurements shown in Figure 2c,d, respectively, demonstrated that the composite film maintained the magnetic characteristics of the embedded hard-magnetic MMPs with a high remanent magnetization and large coercivity value. These properties gave the composite film a strong magnetic-responsiveness and enabled fast, wireless, and efficient magnetically induced shape-morphing for versatile functionalities beyond the pure LCE stimuli-responsiveness.

Next, we characterized the effect of different MMP ratios on the elastic mechanical properties of the composite film via tensile tests on a universal testing machine (Instron 5942, Instron, Norwood, MA, USA). We prepared samples with different MMP concentrations (1:0, 1:4, 1:2, and 1:1 mass ratios of MMPs:LCE) and different uniaxial and splay director fields and measured their stress–strain curves, which are shown in Figure 2e–g. Samples were stretched at a constant displacement rate of 2 mm min^−1^. We repeated the measurement for each configuration on three different samples of the same batch and obtained consistent stress–strain results across all measurements. The stress response of samples with different MMP concentrations but the same director field were within the same order of magnitude, suggesting that the presence of MMPs did not significantly alter the elastic mechanical properties of the LCE matrix.

Although previous studies have investigated the integration of LCE with magnetic nanoparticles (MNPs), especially iron oxide MNPs,^[48,49]^ the introduction of micron-scale ferromagnetic particles, i.e., MMPs, with strong (i.e., “hard”) magnetic properties directly into the LCE matrix remains challenging and presents exciting possibilities. Besides, the locomotion of miniature soft machines in the low Reynolds number regime has not been integrated into a multi-modal locomotion scheme yet. This regime is especially relevant to biological microorganisms and the deployment of miniature machines in medical applications. Within this regime, the viscous forces dominate the inertial forces, which is indicated by a low Reynolds number (≪1), and therefore locomotion can only be achieved by nonreciprocal motions, as explained by the scallop theorem.^[54]^ Helical swimming is an effective and efficient locomotion strategy inside viscous liquids,^[55,56]^ and many studies have reported tiny machines utilizing helical swimming for motility.^[57–59]^ However, none of these helical machines had multi-modal locomotion capability on multiple terrains, such as on surfaces in air and inside viscous liquids, due to the radically different requirements for locomotion strategies resulting from the completely different working environments.

We have shown in the characterization results that the reported film maintains its programmed director field $\vec{n}$ in the LCE matrix and thus its shape-morphing behavior up to a high MMP concentration. Guided by prior works,^[53]^ we programmed director field $\vec{n}$ configurations to induce varying shape-morphing behaviors, such as morphing into a helical shape from a rectangular-shaped film. We utilized this temperature-dependent morphology change to show an untethered soft miniature machine capable of multi-modal locomotion with in situ shape reconfigurability in different working environments, i.e., air and liquid. Being amphibious and motile on both dry surfaces as well as inside a liquid significantly widens the variety of future potential applications of such miniature soft machines. To realize in situ reconfigurability during locomotion, the large shape-morphing of the composite film was triggered by an external stimulus to “self-adapt” the machine body into shapes that are suitable for different locomotion modes in different environments/terrains.

We designed a semicircle curve and a helical shape as the base shapes for two different locomotion modes of walking on surfaces in air and swimming in a viscous media, respectively. We prepared the machine of a rectangular film of 25 μm thickness composite film at a 1:2 mass ratio (MMPs:LCE) with a planar dimension of 1 × 9 mm^2^ and a splay director profile matching that in Figure 2b. We programmed a sinusoidal magnetization profile into the machine body by rolling it on the surface of a glass rod and magnetizing it in a uniform magnetic field of 1.8 T (Figure 3a).^[60]^ It remained flat on a substrate in air at room temperature in the absence of any externally applied magnetic field. We applied a uniform external magnetic field $\vec{B}$ (8 mT) to induce a shape transformation according to the magnetic interactions between the programmed local magnetic moment $\bar{m_{i}}$ of the embedded MMPs and $\vec{B}$, which is described by

(1) $$\vec{\tau_{i}}=\vec{m_{i}}\times \vec{B}$$

where $\vec{\tau_{i}}$ is the resultant magnetic torque corresponding to the respective programmed local $\vec{m_{i}}$. The torque distribution deformed the soft body of the machine into the desired semicircle-like curve. Once in this shape, we superimposed an oscillating $\vec{B}$ varying horizontally with a maximum strength of 4 mT and a minimum of 1 mT. This net $\vec{B}$ made the machine walk on the substrate and move toward the substrate edge. The machine kept walking until it fell off the edge and into the container of the heated liquid (glycerol, ≥99.5%, Sigma-Aldrich, Merck KGaA, dynamic viscosity calculated to be 0.049 N s m^−2^at 70 °C^[61]^). The liquid was preheated to 70 °C, which was found to be sufficient to induce shape-morphing of the composite film to form an overall helical shape. Once the desired helical shape was formed, $\vec{B}$ was rotated in the horizontal plane (4 mT at 3 Hz), causing the machine to rotate with respect to its long axis. This rotatory motion was transferred to translational displacement as explained in previous studies.^[55,56]^ We controlled the machine to move with and against gravity to showcase its motility. Once $\vec{B}$ was removed, the machine sank to the bottom of the container due to its high relative density. The aforementioned procedure is schematically illustrated (Figure 3b) and shown in frames (Figure 3c) taken from Movie S1 (Supporting Information). Although multi-modal locomotion in air and water has been previously demonstrated,^[13]^ the machine always remained as a sheet and employed a combination of a series of minor shape-morphing for different gaits. The change from a “sheet” to a “helix” shown here is drastically different with previously shown shape-morphing and enables very different locomotion modes in two media with vastly different viscosities, i.e., air and glycerol. The viscous environment involved in the reported results is more relevant to future potential biomedical applications of miniature robots. For example, in complex terrains, the walking gait could be used when the machine is first placed on a solid tissue surface for easy deployment and the swimming gait could be used when the machine is actuated to move close to atargeted site submerged in body fluids. An extensive discussion on the effect of the parameters of the helical shape, such as its pitch and number of turns, on the propulsion performance is out of the scope of this work and readily available in the literature.^[55,56]^

Then, we showcased the self-adaptability of the reported material to environmental changes using a slender filament of the composite with a 1:2 mass ratio (MMPs:LCE) and dimensions of 17 mm × 0.13 mm × 25 μm. Its director profile was also a splay field with its planar surface parallel with the aligning direction. We magnetized the filament (1.7 T) to program a homogeneous magnetization profile along its long axis. It was fixed at one end to a glass slide using a glue (Loctite 401 Instant Adhesive, Henkel Corporation), leaving the other end to move freely. We applied a rotating magnetic field of 40 mT using a Halbach array mounted on a step motor. The sample swung back and forth with the rotating magnetic field until a needle heated to around 80 °C was brought into its vicinity. Upon contact, the hot needle triggered a large shape-morphing effect of the filament such that the filament curled and then grasped the needle. The behavior of the soft composite filament in this experiment mimicked the twining motion of vines. This experiment is schematically illustrated and visualized in Figure 4a from the video frames of Movie S2 (Supporting Information).

At last, we demonstrated the potential application of the reported multi-responsive material as reconfigurable magnetic springs for miniature robotics. A magnetic spring utilizes the magnetic interactions between multiple magnetic parts to generate a tunable nonlinear force-displacement profile,^[62,63]^ based on

(2) $$\vec{F}=\nabla (\vec{m}\cdot \vec{B})$$

where $\vec{F}$ is the resultant force corresponding to the magnetic moment $\vec{m}$ in a magnetic field $\vec{B}$. The reported material provides additional in situ reconfigurability for magnetic springs through environmental cues. A proof-of-concept demonstration is presented in Figure 4b. We magnetized a rectangular film with a 1:2 MMPs:LCE mass ratio (16 mm × 8 mm × 40 μm) at 1.8 T to program a uniform magnetic moment $\vec{m}$ perpendicular to its surface. We programmed its director field as a splay formation across its thickness such that out-of-plane bending could be induced by environmental cues. We attached the sample at its center to a circular glass slide mounted on a load cell (25 g, Transducer Techniques). A permanent magnet (3.5 cm diameter and 5 mm thickness, N42, Webcraft GmbH) was placed 1.8 cm below the sample, providing a magnetic field $\vec{B}$ of around 40 mT antiparallel with the sample’s magnetic moment $\vec{m}$. Based on Equation (2), the sample experienced a repulsive force $\vec{F}$ that increased when the sample was pushed toward the magnet. Next, a photothermal deformation of the sample was triggered once it was exposed to UV light (OmniCure Series 2000, Polytec) for 1 min due to the light-responsiveness of the LCE base matrix. The deformation of the sample was retained by the magnetic interactions between the sample and the magnet even after the light was removed. Once deformed, the sample experienced a much smaller $\vec{F}$ from the magnet because its net magnetic moment was reduced. This experiment showed that the composite material enabled in situ reconfigurable force-displacement profiles for magnetic springs with environmental stimuli responsiveness. This photoresponsiveness is enabled by the Disperse Red 1 component in the material. Such photoresponsiveness makes it possible to induce sophisticated behaviors of the reported material via patterned UV illumination in the future.

In summary, we report a composite film that monolithically integrated the respective advantages of a magnetically responsive active soft material with light- and temperature-triggered reversible shape-morphing of an LCE material. The reported composite film shows versatile magnetic controllability enabled by the embedded MMPs and large shape-morphing behaviors thanks to its LCE matrix. Experimental characterizations show that the MMPs and LCE can form optimal monolithic synergy and that the integrated responses of the composite material to multiple stimuli extend the design space of machines in soft robotics, metamaterials, fluidics, and biomedicine. Having multiple control inputs opens up the possibilities of independently actuating individual DOF of a machine, resulting in more sophisticated functionalities, and more precise controls. This additional DOF is especially beneficial in the field of miniature soft machines, which have limited onboard space to accommodate conventional activation and control mechanisms. Example machines have been demonstrated with experimental results that exhibit in situ reconfigurable motility and selfadaptability to changing environments. Such multi-responsive material is promising for potential future applications in smart actuators, sensors and architectural and other surfaces and multifunctional, reconfigurable and physically intelligent wireless soft machines, robots and devices. Throughout the large number of repeated actuations in this study over a period of one year, we have spotted no fatigue or performance deterioration of the reported materials. It suggests a high material stability over time and use, which also agrees with the previous observations in the literature.^[31]^ Composites of LCE and MMPs have the potential to be used for complex designs of stimuli-responsive machines with a wide variety of functionalities.

## Experimental Section

**Figure 1:**
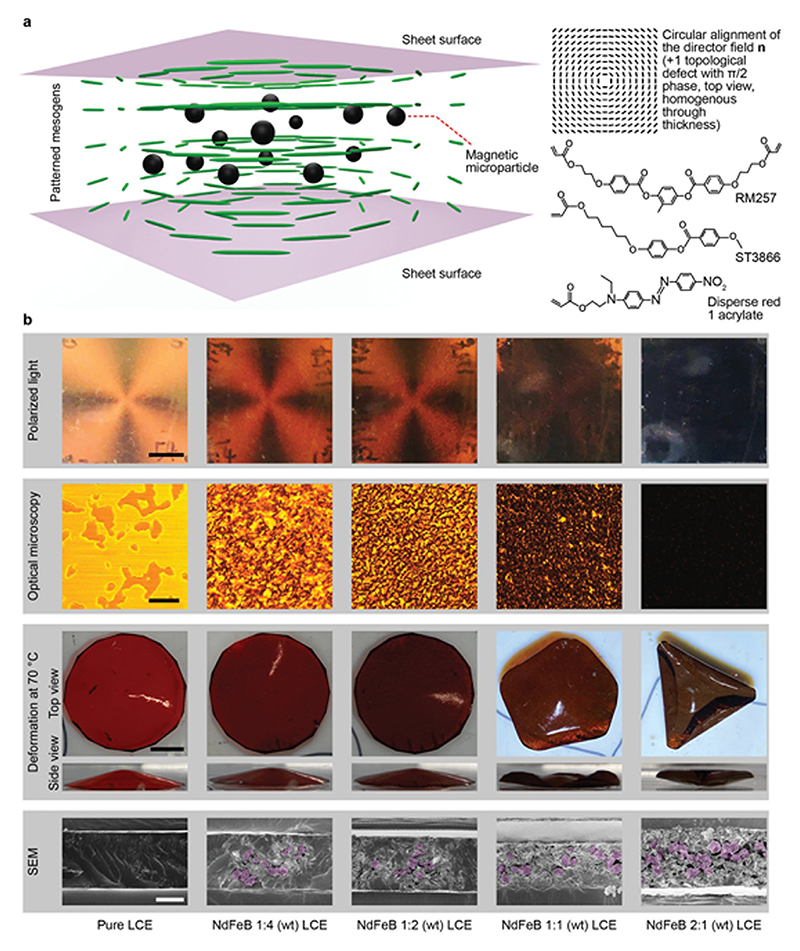
Monolithically integrated magnetic soft liquid crystal elastomer (LCE) composite film for programmable and multiple degrees of freedom (multi-DOF) shape-morphing. a) Schematic illustrations of the proposed composite film for an example circular alignment of the director field of the LCE base matrix. The size and volume ratio of hard NdFeB magnetic microparticles (MMPs) are not to scale. MMPs are conceptually represented by black spheres. b) Schematic and experimental observations of the characteristics of the composite films of different MMP concentrations (0:1, 1:4, 1:2, 1:1, and 2:1) with polarized-light and bright-field optical microscopy. The shape-morphing behaviors of different samples triggered by temperature increase were observed and compared. Cross sections of the samples were observed by scanning electron microscopy (SEM). Pseudocolor was added manually based on sharp edges of MMPs. Scale bars are 0.25 μm for the optical microscopy, 20 μm for the SEM images, and 5 mm for the rest.

*Materials:* Two glass substrates were spin-coated with PI2555 and SE1211 for planar and vertical surface alignment, respectively. The PI2555-coated substrate was rubbed with a cloth unidirectionally to introduce planar alignment along the rubbing direction. These two substrates were assembled together to form a liquid crystal cell with a desired cell gap defined by spherical microparticles. For circular alignment as schematically shown in Figure 1a, two glass substrates coated with PI2555 were circularly rubbed with a cloth and then assembled to form a cell. A mixture of 65 wt% 4-methoxybenzoic acid 4-(6-acryloyloxy-hexyloxy)phenyl ester (ST3866, SYNTHON Chemicals), 32 wt% 1,4-bis[4-(3-acryloyloxypropyloxy)benzoyloxy]-2- methylbenzene (RM257, SYNTHON Chemicals), 2 wt% Disperse Red 1 acrylate (DR1A, Merck KGaA), and 1 wt% photoinitiator 2-benzyl- 2-(dimethylamino)-4’-morpholinobutyrophenone (Merck KGaA) was melted on a 120 °C hot stage for 1 h. The chemical structures of the employed mesogens are illustrated in Figure 1a. Then, MMPs (NdFeB, MQP-15-7, Magnequench, average diameter of 5 μm) were added to the mixture at a selected weight ratio, e.g., 1:2, and uniformly mixed. The mixtures were then filled into liquid crystal cells with capillary force in their isotropic phase (on a 120 °C hot stage) to avoid flow alignment. The liquid crystal cells were then cooled to room temperature slowly, followed by photopolymerization with a UV lamp for 1 h. Finally, the cured liquid crystal elastomer film was separated from glass substrates. Machines were laser cut from the film with the desired geometry designed in AutoCAD (Autodesk Inc.). The machines were then shaped on a glass rod and magnetized in a strong magnetic field generated by a vibrating sample magnetometer (VSM, EZ7, Microsense).^[64]^

*Magnetic Characterization:* Samples of the proposed material were prepared at different MMP concentrations, including 1:4, 1:2, 1:1, and 2:1 mass ratios between MMPs and LCE. The mass values of these samples were measured (Secura225D-1S, Sartorius Lab Instruments GmbH & Co. KG) to be 9.84, 12.64, 11.52, and 16.49 mg, respectively. The samples were fixed onto square glass slides (12 × 12 mm^2^ cover glasses) using enamel (Express Manicure, Maybelline). The glass slides were attached to the VSM and the characterizations were performed at 400 Oe step size with 5 points measured at each setting.

## Supplementary Material

Movie 1

Movie 2

Supplementary material

## Figures and Tables

**Figure 2:**
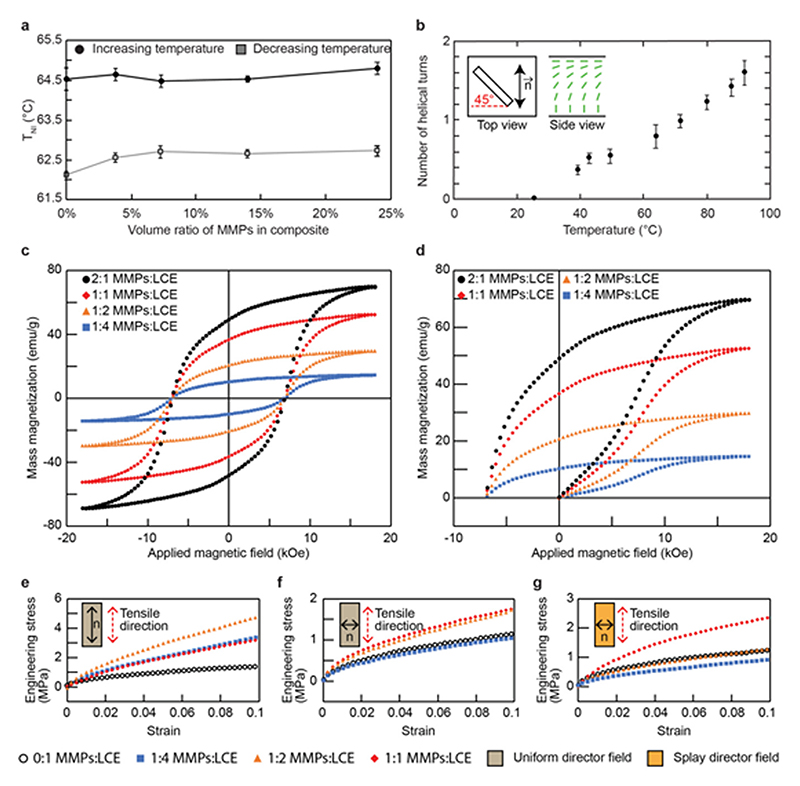
Behavioral and mechanical characterization of the proposed composite material in different MMP concentrations. a) The transition temperature *T*_NI_, between the nematic and isotropic state of the proposed composite material at different MMP concentrations. The value of *T*_NI_ was determined by microscopic observation of liquid monomer textures in both heating and cooling processes with a rate of 5 °C min^−1^. Each column and error bar represent the average and the standard deviation of four measurements, respectively. b) Shape-morphing of the samples (1 mm × 9 mm × 25 pm size film with a 1:2 mass ratio between MMPs and LCE and splay alignment of mesogens across thickness) at different temperatures. Each data point and error bar represent the average and the standard deviation of three measurements, respectively. c) Measured magnetic hysteresis curves (four-quadrant BH curves) of the composite materials at different MMP concentrations. d) Measured initial magnetic curves (BH curves) of the composite materials at different MMP concentrations. e–g) Measured stress–strain curves of the composite materials at different MMP concentrations with different director fields: mesogens were aligned: e) along the tensile direction, f) perpendicular to the tensile direction, and g) perpendicular to the tensile direction with a splay formation across the film thickness.

**Figure 3:**
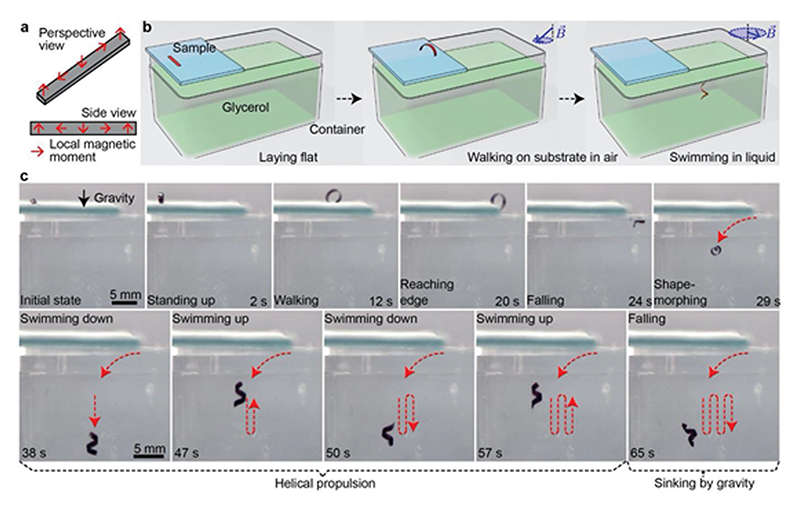
An untethered in situ reconfigurable soft miniature machine that self-adapts to different environments/terrains by exhibiting distinct locomotion modes. a) The pre-programmed magnetization profile views of the sheet-shaped soft machine. b) Schematic illustrations of different locomotion modes on a solid surface in air and inside a viscous fluid exhibited by the proposed machine in response to exerted magnetic fields. c) Video image snapshots from Movie S1 (Supporting Information) of the proposed machine walking on a substrate in air and swimming by helical propulsion inside a viscous liquid. The switching between these two locomotion modes was triggered autonomously by an increase in the environmental temperature (from room temperature to 70 °C).

**Figure 4:**
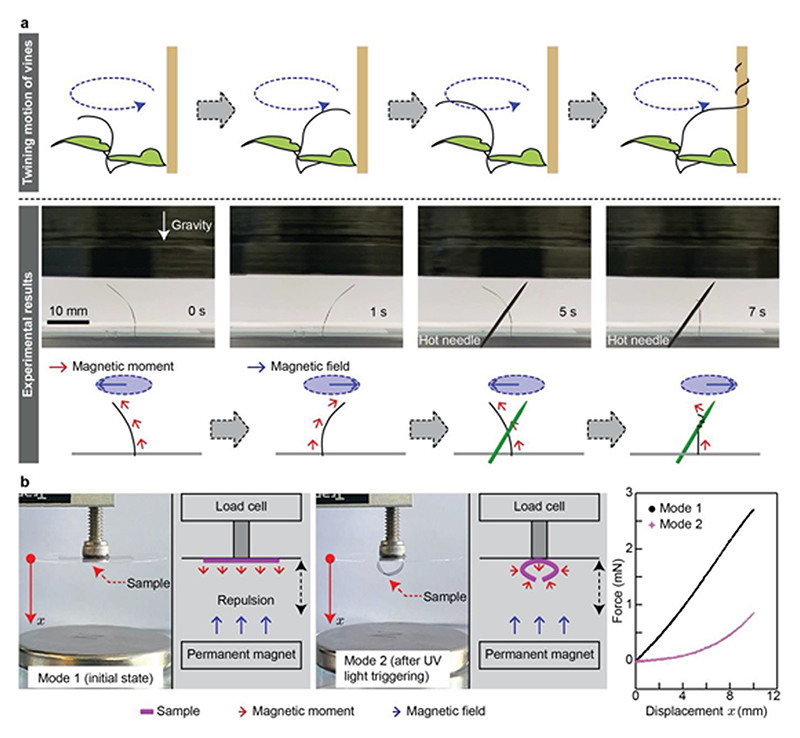
Experimental demonstrations of a vine-plant-inspired filament and a reconfigurable magnetic spring. a) A filament of the proposed composite film twined around a hot needle in its vicinity when it was swaying left and right with the exerted rotating magnetic field. The demonstrated behavior of the composite film mimicked the twining motion of the climbing vine plants (see Movie S2 in the Supporting Information). b) A proof-of-concept magnetic spring with reconfigurable damping profiles. The sample made of the proposed composite film resisted the movement along x-axis (Mode 1). After being exposed to UV light, the sample curled and the deformation persisted once the light was removed. The deformed sample exhibited a different resistance curve along x-axis (Mode 2). Note that the force–displacement profiles for both modes start from 0 to 10 mm and then come back from 10 to 0 mm. The preceding and receding curves overlap with each other, suggesting no observable hysteresis.

## References

[1] Gelebart AH, Mulder DJ, Varga M, Konya A, Vantomme G, Meijer EW, Selinger RLB, Broer DJ (2017). Nature.

[2] Zeng H, Wani OM, Wasylczyk P, Kaczmarek R, Priimagi A (2017). Adv Mater.

[3] Shahsavan H, Aghakhani A, Zeng H, Guo Y, Davidson ZS, Priimagi A, Sitti M (2020). Proc Natl Acad Sci USA.

[4] Tabrizi M, Ware TH, Shankar MR (2019). ACS Appl Mater Interfaces.

[5] Boothby JM, Ware TH (2017). Soft Matter.

[6] Ware TH, McConney ME, Wie JJ, Tondiglia VP, White TJ (2015). Science.

[7] Kotikian A, McMahan C, Davidson EC, Muhammad JM, Weeks RD, Daraio C, Lewis JA (2019). Sci Rob.

[8] Guin T, Settle MJ, Kowalski BA, Auguste AD, Beblo RV, Reich GW, White TJ (2018). Nat Commun.

[9] Zhang J, Diller E (2016). Smart Mater Struct.

[10] Zhang J, Diller E (2018). Soft Rob.

[11] Zhang J, Onaizah O, Middleton K, You L, Diller E (2017). IEEE Rob Autom Lett.

[12] Zhang YF, Ng CJX, Chen Z, Zhang W, Panjwani S, Kowsari K, Yang HY, Ge Q (2019). Adv Mater Technol.

[13] Hu W, Lum GZ, Mastrangeli M, Sitti M (2018). Nature.

[14] Ren Z, Hu W, Dong X, Sitti M (2019). Nat Commun.

[15] Guo Y, Shahsavan H, Sitti M (2020). Adv Mater.

[16] Davidson ZS, Shahsavan H, Aghakhani A, Guo Y, Hines L, Xia Y, Yang S, Sitti M (2019). Sci Adv.

[17] Terentjev EM (1999). J Phys : Condens Matter.

[18] Fusco S, Huang H-W, Peyer KE, Peters C, Häberli M, Ulbers A, Spyrogianni A, Pellicer E, Sort J, Pratsinis SE, Nelson BJ (2015). ACS Appl Mater Interfaces.

[19] Xie T, Xiao X, Cheng YT (2009). Macromol Rapid Commun.

[20] Tang J, Zhou Y, Wan L, Huang F (2018). Macromol Rapid Commun.

[21] Yuan C, Ding Z, Wang TJ, Dunn ML, Qi HJ (2017). Smart Mater Struct.

[22] Guo Y, Shahsavan H, Sitti M (2020). Adv Opt Mater.

[23] Kotikian A, Truby RL, Boley JW, White TJ, Lewis JA (2018). Adv Mater.

[24] Kim H, Boothby JM, Ramachandran S, Lee CD, Ware TH (2017). Macromolecules.

[25] Küpfer J, Finkelmann H (1991). Makromol Chem Rapid Commun.

[26] Mol GN, Harris KD, Bastiaansen CWM, Broer DJ (2005). Adv Funct Mater.

[27] Yu Y, Nakano M, Ikeda T (2003). Nature.

[28] Barnes M, Verduzco R (2019). Soft Matter.

[29] Zhang C, Lu X, Fei G, Wang Z, Xia H, Zhao Y (2019). ACS Appl Mater Interfaces.

[30] Saed MO, Ambulo CP, Kim H, De R, Raval V, Searles K, Siddiqui DA, Cue JMO, Stefan MC, Shankar MR, Ware TH (2019). Adv Funct Mater.

[31] Xiao Y, Jiang Z, Tong X, Zhao Y (2019). Adv Mater.

[32] Zeng H, Wasylczyk P, Parmeggiani C, Martella D, Burresi M, Wiersma DS (2015). Adv Mater.

[33] Van Oosten CL, Bastiaansen CWM, Broer DJ (2009). Nat Mater.

[34] Wani OM, Zeng H, Priimagi A (2017). Nat Commun.

[35] Martella D, Nocentini S, Nuzhdin D, Parmeggiani C, Wiersma DS (2017). Adv Mater.

[36] Schuhladen S, Preller F, Rix R, Petsch S, Zentel R, Zappe H (2014). Adv Mater.

[37] Pilz da Cunha M, Kandail HS, den Toonder JMJ, Schenning APHJ (2020). Proc Natl Acad Sci USA.

[38] Ford MJ, Ambulo CP, Kent TA, Markvicka EJ, Pan C, Malen J, Ware TH, Majidi C (2019). Proc Natl Acad Sci USA.

[39] Yu L, Peng R, Rivers G, Zhang C, Si P, Zhao B (2020). J Mater Chem A.

[40] Sitti M (2017). Mobile Microrobotics.

[41] Du X, Cui H, Xu T, Huang C, Wang Y, Zhao Q, Xu Y, Wu X (2020). Adv Funct Mater.

[42] Huang HW, Sakar MS, Petruska AJ, Pané S, Nelson BJ (2016). Nat Commun.

[43] Ze Q, Kuang X, Wu S, Wong J, Montgomery SM, Zhang R, Kovitz JM, Yang F, Qi HJ, Zhao R (2019). Adv Mater.

[44] Miyashita S, Diller E, Sitti M (2013). Int J Rob Res.

[45] Pilz da Cunha M, Foelen Y, van Raak RJH, Murphy JN, Engels TAP, Debije MG, Schenning APHJ (2019). Adv Opt Mater.

[46] Pilz da Cunha M, Foelen Y, Engels TAP, Papamichou K, Hagenbeek M, Debije MG, Schenning APHJ (2019). Adv Opt Mater.

[47] Herrera-Posada S, Mora-Navarro C, Ortiz-Bermudez P, Torres-Lugo M, McElhinny KM, Evans PG, Calcagno BO, Acevedo A (2016). Mater Sci Eng C.

[48] Song HM, Kim JC, Hong JH, Lee YB, Choi J, Lee JI, Kim WS, Kim JH, Hur NH (2007). Adv Funct Mater.

[49] Kaiser A, Winkler M, Krause S, Finkelmann H, Schmidt AM (2009). J Mater Chem.

[50] Riou O, Zadoina L, Lonetti B, Soulantica K, Mingotaud AF, Respaud M, Chaudret B, Mauzac M (2012). Polymers.

[51] Yao Y, Waters JT, Shneidman AV, Cui J, Wang X, Mandsberg NK, Li S, Balazs AC, Azienberg J (2018). Proc Natl Acad Sci USA.

[52] Pishnyak OP, Tang S, Kelly JR, Shiyanovskii SV, Lavrentovich OD (2007). Phys Rev Lett.

[53] White TJ, Broer DJ (2015). Nat Mater.

[54] Purcell EM (1977). Am J Phys.

[55] Abbott J, Peyer KE, Lagomarsino MC, Zhang L, Dong L, Kaliakatsos IK, Nelson BJ (2009). Int J Rob Res.

[56] Barbot A, Decanini D, Hwang G (2016). Sci Rep.

[57] Qiu F, Fujita S, Mhanna R, Zhang L, Simona BR, Nelson BJ (2015). Adv Funct Mater.

[58] Ceylan H, Yasa IC, Yasa O, Tabak AF, Giltinan J, Sitti M (2019). ACS Nano.

[59] Zhang L, Abbott J, Dong L, Kratochvil B, Bell D, Nelson BJ (2009). Appl Phys Lett.

[60] Diller E, Zhuang J, Lum G, Edwards M, Sitti M (2014). Appl Phys Lett.

[61] Volk A, Kähler CJ (2018). Exp Fluids.

[62] Woodward MA, Sitti M (2019). IEEE Trans Rob.

[63] Woodward MA, Sitti M (2018). IEEE Trans Magn.

[64] Lum GZ, Ye Z, Dong X, Marvi H, Erin O, Hu W, Sitti M (2016). Proc Natl Acad Sci USA.

