# Supplementary material for: Liquid Crystal Elastomer-Based Magnetic Composite Films for Reconfigurable Shape-Morphing Soft Miniature Machines
Source: Adv Mater. Author manuscript; Available in PMC 2021 Mar 29. (PMC7610459; doi:10.1002/adma.202006191)
Supplement: Supplementary material [file EMS119914-supplement-Supplementary_material.pdf]

© 2021 Wiley-VCH GmbH

ADVANCED MATERIALS

Supporting Information

for *Adv. Mater.*, DOI: 10.1002/adma.202006191

Liquid Crystal Elastomer-Based Magnetic Composite Films for Reconfigurable Shape-Morphing Soft Miniature Machines

*Jiachen Zhang, Yubing Guo, Wenqi Hu, Ren Hao Soon, Zoey S. Davidson, and Metin Sitti**

Supporting Information

Liquid crystal elastomer-based magnetic composite films for reconfigurable shape-morphing soft miniature machines

Jiachen Zhang[†]*, Yubing Guo*[†]*, Wenqi Hu*[†]*, Ren Hao Soon, Zoey S. Davidson and Metin Sitti*

[†] Equally contributing first authors

We characterized uniform alignment of an MMP-doped LCE film with a mass ratio of 1:2 (MMPs:LCE) and presented the optical microscope images in **Figure S1**. From the bight field image (Figure S1a), we observed that MMPs aggregated into certain regions and blocked the light. Regions without MMPs exhibited large light intensity contrast before and after rotation of the LCE film by 45°, as can be seen by comparing Figure S1b, c, indicating good alignment of LCE in these regions.

In order to compare uniform alignment quality of LCE doped with different MMP concentrations, we measured transmission spectra of different samples sandwiched between either parallel polarizers or crossed polarizers. We fixed the LCE director field to be along optical axis of one polarizer and rotated another polarizer to align its optical axis either parallel (for parallel polarizers) or perpendicular (for crossed polarizers) to LCE director field. The recorded transmission spectra are presented in **Figure S2**a and b, which indicate that transmission decreases with increasing MMP concentrations for both parallel polarizers and crossed polarizers due to non-transparency of MMPs. We then calculated ratios between transmission with parallel polarizers and transmission with crossed polarizers for different samples and presented the results in Figure S2c. Basically, higher ratio corresponds to better uniform alignment of LCE. We observed that alignment quality decreases gradually as MMP increases from 0 to 1:2 mass ratio; and the quality drops abruptly when MMP increases to 1:1 mass ratio. Note that even though the alignment quality is worse than those with lower MMP concentrations, sample with 2:1 mass ratio of MMPs still has maximum polarization ratio of around 5.

Next, we characterized the Fourier-transform infrared spectroscopy (FTIR, Tensor II, Bruker Optik GmbH) of thin-film samples with 25 μm thickness and a 1:2 mass ratio between MMPs and LCEs, which is the configuration used by all the functional demonstrations. The samples were prepared with different UV photo-crosslinking time: 0, 5, 10, 30, and 60 minutes. A comparison between the spectra of samples with 0 and 60 minutes cross-linking is shown in **Figure S3**a. Two peaks identified at 1634 and 984 cm^{-1} correspond to the C−H bond stretching vibration in C=C and the =C−H bond out-of-plane vibration in $RCH=CH_2$ of the acrylate end group, respectively. The significant reduction of these two peaks indicates the removal of these bonds during photo-crosslinking. In addition, samples prepared with 5, 10, and 30 minutes of photo-crosslinking time were characterized and results are presented in Figure S3b, together with the spectrum of a reference sample with no MMPs and photo-crosslinked for 60 minutes. The spectra of these sample overlap with each other with some minor measurement errors, suggesting the crosslinking finishes within 5 minutes and the 60-minute period of time utilized in this work ensures full crosslinking of the samples. The results also suggest the existence of MMPs in these samples does not impede the crosslinking of the LCEs and therefore does not detrimentally affect the corresponding stimuli-responsiveness. Although the discrete MMPs disrupt microscopic homogeneity, as observed in **Figure 1**b, the samples experimentally exhibit macroscopic homogeneous shape-morphing behavior at the size scale concerned in this study,

i.e., several hundreds of microns to several millimeters, suggesting a good overall crosslinking and behavioral homogeneity across the material.

Moreover, we characterized the in-plane contracting and expanding deformation of the reported composite material with different MMP concentrations, and results are shown in **Figure S4**. We prepared samples with dimensions of 5 mm × 5 mm × 40 µm and submerged them in water baths. We took top-view images of the samples while raising the temperature. We measured the dimensional changes of the samples and calculated the strain changes along the directions parallel and perpendicular to the direction field. We tested four samples from the same batch for each MMP concentration. Results in Figure S4b confirms that the material maintained the programmed director field up to a very high MMP concentration.

We characterized the reported material at high temperatures, which are beyond the designed working range from room temperature to a moderate (<100 °C) temperature. Samples were prepared to have a 25 µm thickness and a 1:2 mass ratio between MMPs and LCEs, which is the configuration used by the functional demonstrations. A sample was observed at polarized optical microscopy (POM) up to 140 °C and no sign of phase transition was spotted, see **Figure S5**a. Another two rectangular samples were placed on a hotplate and heated up to 300 °C. The samples first formed their designated 3D shape in the designed temperature range (at 60 °C). With the temperature being further increased, the samples deformed more and eventually different parts of their bodies crashed into each other, forming tightly packed lumps. In extreme cases, the samples even broke itself into pieces since they could not stand the self-compression. When cooled down, the broken samples were unable to recover their initial shapes. These results are shown in Figure S5b and suggest that the reported material works as expected at a temperature range way below its transition temperature. A temperature reaching or surpassing the transition temperature will cause over-deformation and therefore is undesired.

Finally, we characterized the durability of the reported material over cyclic actuations. A sample prepared with a 25 µm thickness and a 1:2 mass ratio between MMPs and LCEs was repeated placed on and removed from a hotplate set to 65 °C for 50 times. The room temperature is 22 °C. The observed shape-morphing of the material is shown in **Figure S6**. The sample was initially in a flat shape and formed a complete helix when being thermally triggered. When the sample was removed from the hotplate and cooled down to room temperature, it maintained a small degree of residual shape from its previous deformation, which is a result of the slight plastic distortion that is common in LCEs. Throughout the 50 times of repeated actuation, the sample exhibited consistent shape-morphing behavior and assumed nearly identical shapes in each cycle. After the test, the sample restored to its initial flat shape upon gentle touch, which compensated the slight plastic distortion. This test experimentally shows the good durability and consistency of the reported material under repeated actuations. This excellent durability could be attributed to the superior durability of LCEs in general and the experimentally observed fact that MMPs do not disrupt the crosslinking of the LCE under the setting in this work. As for the durability of MMPs, the employed NdFeB particles are widely utilized as permanent magnetic materials and their magnetic properties do not deteriorate unless being re-magnetized using strong magnetic field (over several hundreds of milliTesla) or being heated to its Curie temperature at around 300 °C, both of which are way higher than the designed working range of this study.

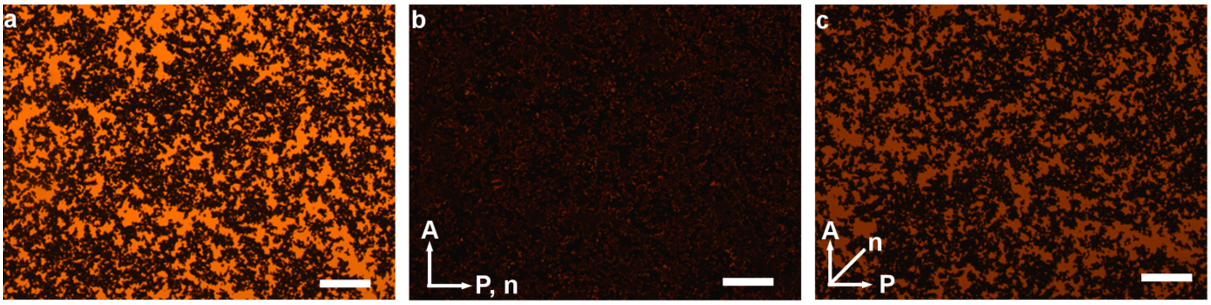

Figure S1. Optical microscope images of MMP-doped LCE with uniform surface alignment. a) Bright field image of MMP-doped LCE. **b, c)** Cross-polarized optical microscope images of MMP-doped LCE with director field along optical axis of polarizer (a) or **45°** to this axis (b). P, A, and n represent optical axis of polarizer, optical axis of analyzer, and LC director. Scale bars are 200 μm for all the figures.

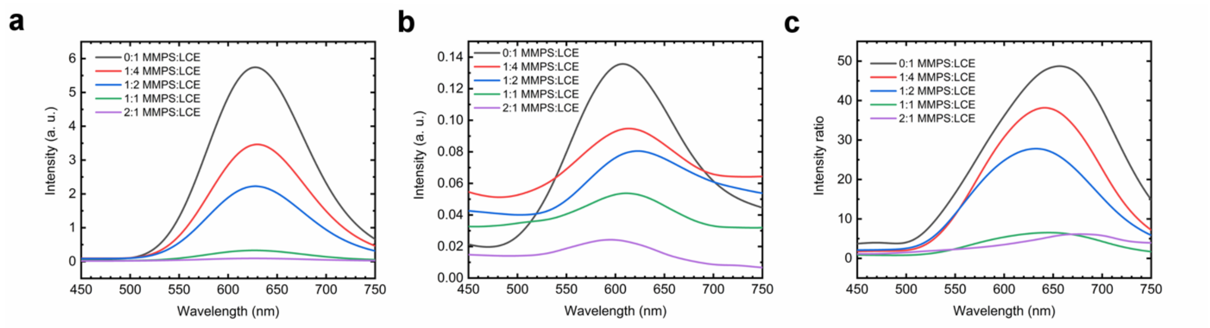

Figure S2. Optical characterization of uniform alignment for LCE doped with different MMP concentrations. a, b) Transmission spectra of samples sandwiched between parallel polarizers (a) and crossed polarizers (b). **c)** Ratio between transmission with parallel polarizers and transmission with cross polarizers for different samples.

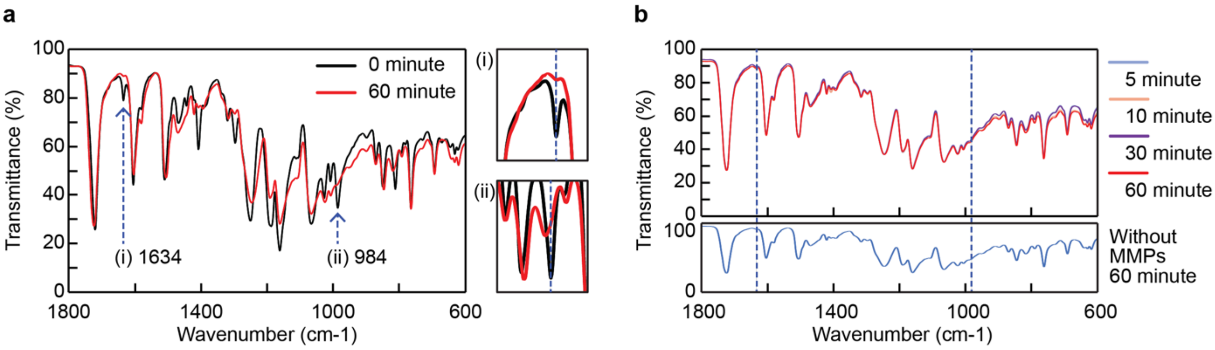

Figure S3. Fourier-transform infrared spectroscopy (FTIR) characterization of samples prepared with different UV cross-linking time and 1:2 MMP:LCE mass ratio. a) Comparison between the spectra of samples prepared with 0 and 60 minutes of UV crosslinking time. Two peaks identified at 1634 and 984 cm^{-1} correspond to the C−H bond stretching vibration in C=C and the =C−H bond out-of-plane vibration in $RCH{=}CH_2$ of the acrylate end group, respectively. Zoomed-in comparisons of the two peaks are shown in (i) and (ii), respectively. **b)** Comparison between the spectra of samples prepared with 5, 10, 30, and 60 minutes of UV crosslinking time. The spectrum of a reference sample (no MMPs, photo-crosslinked for 60 minutes) is shown at the bottom. The two peaks considered in (a) are marked out by dashed lines.

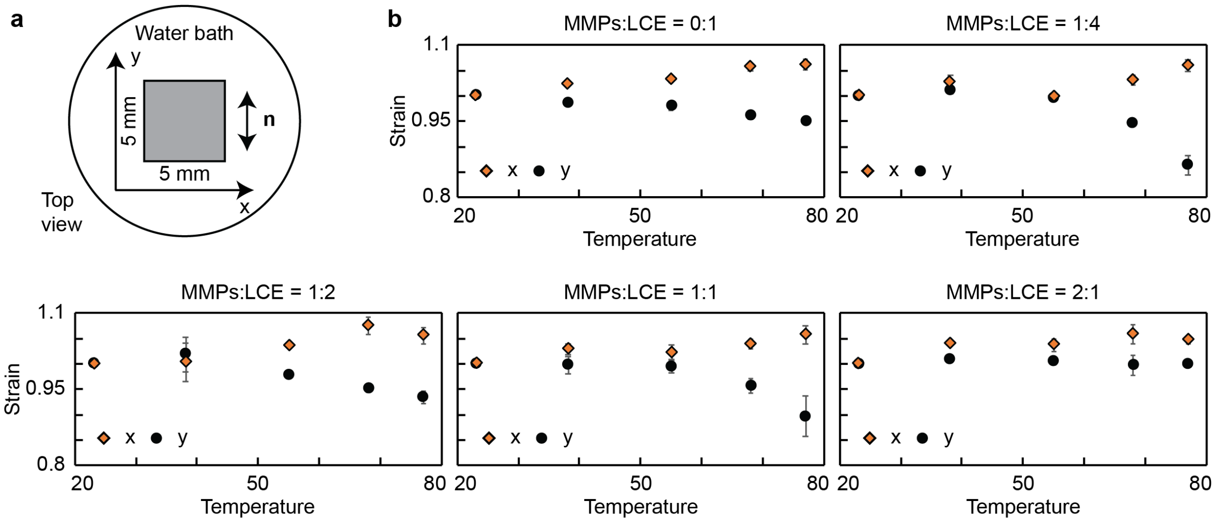

Figure S4. Strain characterization of the reported composite material in terms of temperature changes with different MMP concentrations. a) Schematic illustration of the characterization experimental setup. **b)** Experimental results of the strain variations of samples with different MMP concentrations in response to temperature changes. Each data point and error bar represent the average and standard deviation of four measurements, respectively.

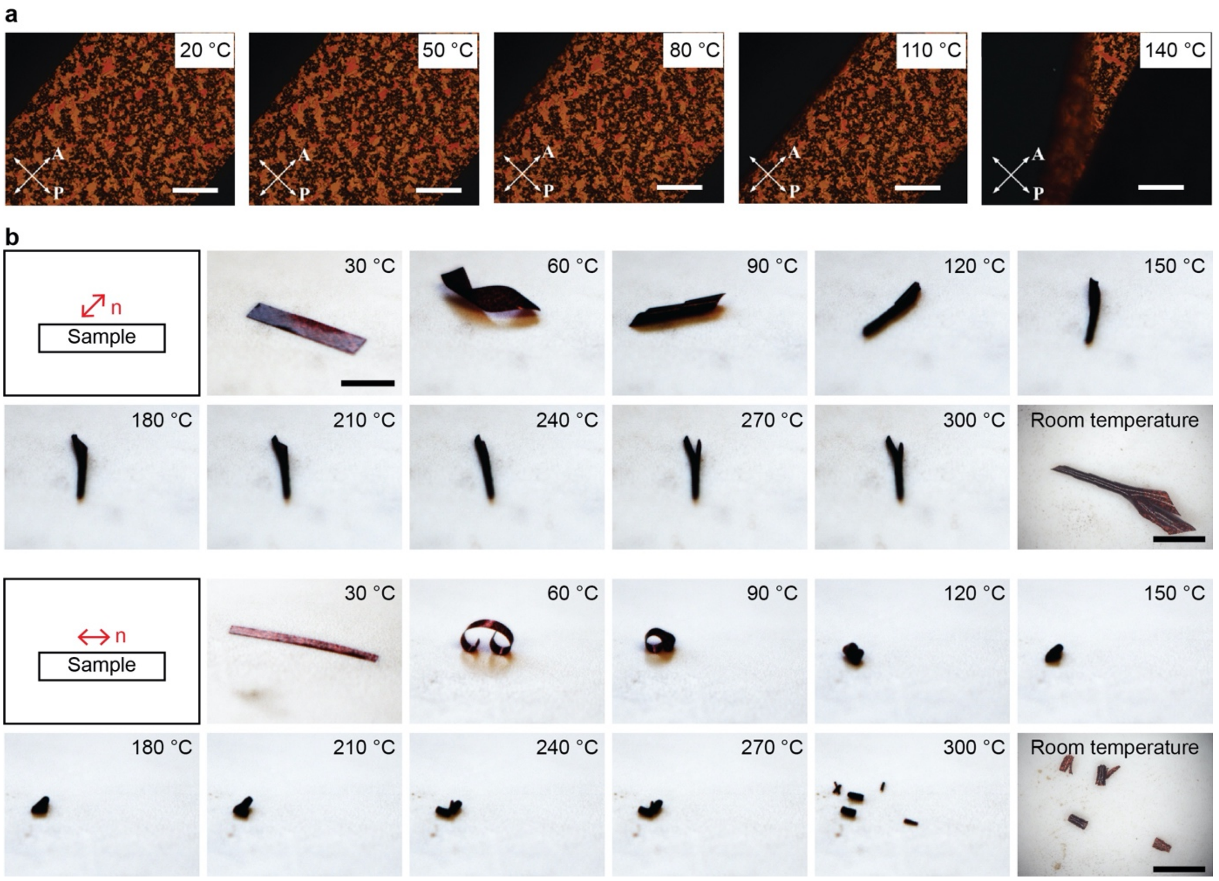

Figure S5. Characterizations of the material at high temperatures. a) Observations of a sample at polarized optical microscopy (POM) at temperatures from 20 to 140 °C. The blur in the last frame is caused by the sample breaking loose of the tape fixture. **b)** Observations of the shape-morphing behaviors of two samples at temperatures from 30 to 300 °C. Scalar bars are 500 µm for (a) and 300 µm for (b).

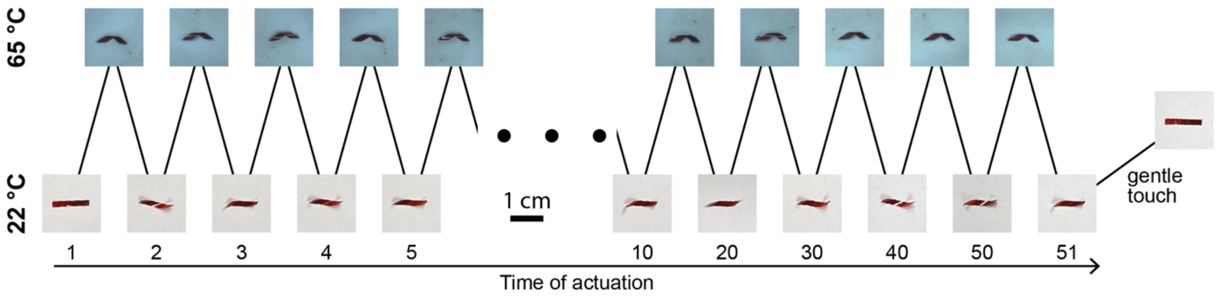

Figure S6. Durability characterizations of the material in cyclic actuations. The sample was repeated placed on and removed from a hotplate set to 65 °C for 50 times. When removed from the hotplate, the sample was cooled down to room temperature (22 °C) and observed.

Supporting Movies

Movie S1. A millimeter-scale untethered machine exhibits in situ reconfigurable locomotion mode using the proposed composite film. The machine is activated by a magnetic field and walks on a dry substrate in air. When it goes into viscous liquid, the machine takes a helical shape, induced by the temperature-responsiveness of its LCE base, and rotates to move in a corkscrew gait.

Movie S2. A vine plant-inspired filament mode of the proposed composite film exhibits adaptability to its environment. The filament moves back and forth, mimicking the rotating motion of a vine plant. When a hot needle is brought close to the vicinity, the filament deforms and grasps the needle, just like the twining phenomenon of a vine plant. A control trial with a cool needle is also presented side-by-side with the experimental trial.
